# Commentary: An Elusive and Deceptive Tachycardia

**DOI:** 10.19102/icrm.2017.080201

**Published:** 2017-02-15

**Authors:** E. Kevin Heist


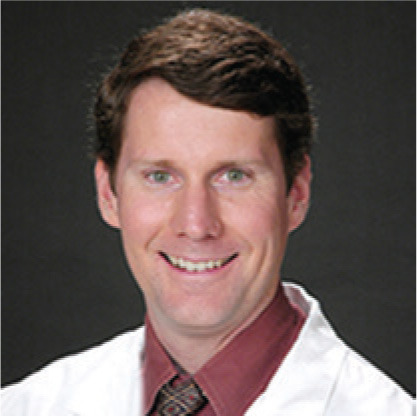


In the electrophysiology (EP) field, many of us have become accustomed to pattern recognition, and during EP procedures, hoofbeats are usually caused by horses and not zebras. There are zebras around in all of our EP laboratories, however, and, thus, staying attuned to them can make all the difference in correctly interpreting an unusual arrhythmia and achieving the completion of a successful procedure.

In this particular case, a wide complex tachycardia was induced with atrioventricular (AV) dissociation during tachycardia, with no ventriculoatrial (VA) conduction evident prior to the initiation of tachycardia. These findings (i.e. wide complex tachycardia, AV dissociation during tachycardia and no VA conduction at baseline) would lead most of us to initially place ventricular tachycardia at positions 1, 2, and 3 of our differential diagnosis. In this case, however, the author astutely noted the patient’s history of narrow complex tachycardia on Holter monitoring, as well as the relatively narrow QRS and very short/negative HV interval during the tachycardia, which pointed them to the correct diagnosis of a nodo-fascicular/ventricular pathway supporting the tachycardia. Unfortunately, in this particular case, the tachycardia was not sufficiently inducible and sustained to allow for full maneuvers to determine mechanism; however, the tachycardia was successfully ablated with slow pathway ablation.

Many of us in EP might go through our entire careers and never see a nodo-fascicular or nodo-ventricular pathway-mediated tachycardia in our EP laboratory. Others might occasionally actually have one of these cases in their EP laboratory, but not “see it” or realize its significance, because we don’t think of it in our differential diagnosis. So remember, zebras are out there!

Sincerely,

E. Kevin Heist, MD, PHD, FACC, FHRS

Associate Professor of Medicine

Cardiac Arrhythmia Service

kheist@mgh.harvard.edu

Harvard Medical School, Massachusetts General Hospital

GRB 109, 55 Fruit Street

Boston, MA 02114

